# Bevacizumab Exacerbates Paclitaxel-Induced Neuropathy: A Retrospective Cohort Study

**DOI:** 10.1371/journal.pone.0168707

**Published:** 2016-12-19

**Authors:** Ayumu Matsuoka, Osamu Maeda, Takefumi Mizutani, Yasuyuki Nakano, Nobuyuki Tsunoda, Toyone Kikumori, Hidemi Goto, Yuichi Ando

**Affiliations:** 1 Department of Clinical Oncology and Chemotherapy, Nagoya University Hospital, Nagoya, Japan; 2 Department of Gastroenterology and Hepatology, Nagoya University Graduate School of Medicine, Nagoya, Japan; 3 Department of Clinical Oncology, Japanese Red Cross Nagoya Daiichi Hospital, Nagoya, Japan; 4 Division of Surgical Oncology, Department of Surgery, Nagoya University Graduate School of Medicine, Nagoya, Japan; 5 Department of Breast and Endocrine Surgery, Nagoya University Hospital, Nagoya, Japan; Medical University Innsbruck, AUSTRIA

## Abstract

**Background:**

Bevacizumab (BEV), a humanized anti-vascular endothelial growth factor (VEGF) monoclonal antibody, enhances the antitumor effectiveness of paclitaxel (PTX)-based chemotherapy in many metastatic cancers. A recent study in mice showed that VEGF receptor inhibitors can interfere with the neuroprotective effects of endogenous VEGF, potentially triggering the exacerbation of PTX-induced neuropathy. In clinical trials, exacerbation of neuropathy in patients who received PTX combined with BEV (PTX+BEV) has generally been explained by increased exposure to PTX owing to the extended duration of chemotherapy. We investigated whether the concurrent use of BEV is associated with the exacerbation of PTX-induced neuropathy.

**Methods:**

Female patients with breast cancer who had received weekly PTX or PTX+BEV from September 2011 through May 2016 were studied retrospectively. PTX-induced neuropathy was evaluated at the same time points (at the 6^th^ and 12^th^ courses of chemotherapy) in both cohorts. A multivariate Cox proportional-hazards model was used to assess the independent effect of BEV on the time to the onset of neuropathy.

**Results:**

A total of 107 patients (median age, 55 years; range, 32–83) were studied. Sixty-one patients received PTX as adjuvant chemotherapy, 23 received PTX for metastatic disease, and 23 received PTX+BEV for metastatic disease. Peripheral sensory neuropathy was worse in patients who received PTX+BEV than in those who received PTX alone: at the 6^th^ course, Grade 0/1/2/3 = 4/13/4/0 vs. 25/42/6/0 (*P* = 0.095); at the 12^th^ course, 2/3/11/3 vs. 7/30/23/2 (*P* = 0.016). At the 12^th^ course, the incidence of Grade 2 or higher neuropathy was significantly higher in patients treated with PTX+BEV than in those treated with PTX alone (74% vs. 40%; *P* = 0.017). In multivariate analysis, BEV was significantly associated with an increased risk of neuropathy (HR 2.32, 95% CI 1.21–4.44, *P* = 0.012).

**Conclusions:**

The concurrent use of BEV could worsen PTX-induced neuropathy in patients with breast cancer.

## Introduction

Bevacizumab (BEV), a recombinant humanized monoclonal antibody against vascular endothelial growth factor (VEGF), enhances the antitumor effectiveness of standard chemotherapy in various metastatic cancers, including cancer of the breast, colon, lung and ovary [1]. In patients with metastatic breast cancer, BEV combined with paclitaxel (PTX) improves progression-free survival (PFS) and overall response rate in association with the increasing incidence of adverse events [2, 3]. BEV causes various class side effects, such as hypertension, proteinuria, hemorrhage, gastrointestinal perforation, wound-healing complications, and thromboembolism [4]. Moreover, in clinical trials, non-BEV-related toxic effects were also augmented in the BEV combination arm, which has been generally attributed to the longer duration of effective chemotherapy [4].

PTX commonly causes peripheral sensory neuropathy in a cumulative, dose-dependent manner [5, 6]. In previous clinical trials, the incidence of neuropathy was reported to be higher in patients who had received PTX combined with BEV (PTX+BEV) than in those who had received PTX alone [2]. This exacerbation of neuropathy has generally been explained by the prolonged exposure to PTX owing to the extended duration of chemotherapy [3]. BEV is generally considered to be unrelated to peripheral neuropathy [1, 4]. However, it is unclear whether the concurrent use of BEV is associated with the exacerbation of PTX-induced neuropathy. In this study, we retrospectively compared peripheral sensory neuropathy between patients who had received PTX and those who had received PTX+BEV during the same time frame.

## Materials and Methods

### Ethics Statement

This study was approved by the Institutional Review Board and Ethics Committee of Nagoya University Hospital and Japanese Red Cross Nagoya Daiichi Hospital. The study was conducted in accordance with the principles of the Declaration of Helsinki. No informed consent was required for this retrospective observational study, and patient records and information were anonymized and de-identified before analysis.

### Patients

Japanese female patients with breast cancer who had received PTX (PTX 80 mg/m^2^ weekly) or PTX+BEV (PTX 80 mg/m^2^ on days 1, 8, and 15 combined with BEV 10 mg/kg on days 1 and 15 every 4 weeks) in Nagoya University Hospital or Japanese Red Cross Nagoya Daiichi Hospital were studied. Eligible patients had to (1) be 20 years of age or older; (2) be treated with PTX or PTX+BEV; (3) have a histologically confirmed diagnosis of breast cancer; and (4) have been evaluated for peripheral sensory neuropathy according to the Common Terminology Criteria for Adverse Events (CTCAE), version 4.0 (Table 1). In both hospitals, adverse events were assessed by medical oncologists and well-trained nurses who were specialized in cancer treatment and care, using standardized check-list.

**Table 1:** Peripheral sensory neuropathy according to CTCAE, version 4.0.

	Grade 1	Grade 2	Grade 3
Peripheral sensory neuropathy	Asymptomatic; loss of deep tendon reflexes or paresthesia	Moderate symptoms; limiting instrumental ADLs^†^	Severe symptoms; limiting self-care ADLs^‡^

†Preparing meals, shopping for groceries or clothes, using the telephone, managing money, etc.

‡Bathing, dressing and undressing, using the toilet, taking medications, and not being bedridden.

Abbreviations: ADLs, activities of daily living; CTCAE, Common Terminology Criteria for Adverse Events.

Patients were excluded if they had (1) a medical history of peripheral neuropathy; (2) known risk factors for peripheral neuropathy, such as diabetes mellitus, renal failure, severe liver impairment, hypothyroidism, or alcoholism; (3) brain or central nervous system metastases; (4) previous use of PTX-based chemotherapy; (5) concurrent use of trastuzumab; or (6) concurrent radiation therapy. We excluded patients with concurrent use of trastuzumab because of potential neurotoxic or neuroprotective effects of trastuzumab, if any. We also excluded patients who received concurrent radiation therapy, which was administered in another clinical trial.

### Study Design

In Japan, BEV was approved for the indication of metastatic breast cancer in September 2011. This retrospective cohort study thus included patients who had received PTX or PTX+BEV after September 2011.

The study endpoint was to investigate whether the concurrent use of BEV was associated with the exacerbation of PTX-induced neuropathy. Peripheral sensory neuropathy evaluated according to the CTCAE was compared between the cohorts at the same time points (at the 6^th^ and 12^th^ courses of chemotherapy). Peripheral neuropathy of Grade 2 or higher is clinically important, because it can lead to dose reduction, temporary cessation of treatment, or switching to less neurotoxic regimens [6, 7]. Thus, the incidence of Grade 2 or higher neuropathy and the time to the onset of Grade 2 neuropathy were also compared between the cohorts.

### Statistical Analysis

Patient characteristics are summarized using descriptive statistics. Continuous variables are expressed as medians (range) because of non-normality of the distributions as confirmed by the Shapiro-Wilk test. Categorical variables are expressed as frequencies (percentages). The statistical significance of differences in baseline characteristics between cohorts (PTX vs. PTX+BEV) was assessed with the Mann-Whitney test for continuous variables and the chi-square test or Fisher’s exact test for categorical variables, as appropriate. Peripheral sensory neuropathy according to the CTCAE grade was compared between the cohorts at the 6^th^ and 12^th^ courses of chemotherapy using the Mann-Whitney test. The incidence of neuropathy of Grade 2 or higher at the 6^th^ and 12^th^ courses was also compared between the cohorts using the chi-square test or Fisher’s exact test, as appropriate. Associations between the cohorts (PTX vs. PTX+BEV) and the time to the onset of neuropathy of Grade 2 or higher were estimated using the Kaplan-Meier method and compared using the log-rank test. Data on patients without neuropathy of Grade 2 or higher at the time of the analysis and on those who discontinued the treatment were censored. A multivariate analysis using a Cox proportional-hazards model was performed to assess the independent effects of the regimens (PTX vs. PTX+BEV) on the time to the onset of neuropathy of Grade 2 or higher after adjustment for baseline characteristics and potential confounding factors, such as previous use of docetaxel [6]. After selection of covariates in univariate analyses (*P*<0.20), multivariate analysis was performed. The hazard ratios (HRs) and 95% confidence interval (CI) were calculated. In addition, a subgroup analysis limited to patients with metastatic disease who received or did not receive BEV was also performed. All calculations were performed using the SPSS software package, version 23 (SPSS Inc., Chicago, Illinois, USA), and two-sided *P* values < 0.05 were considered to indicate statistical significance.

## Results

**Fig 1:**
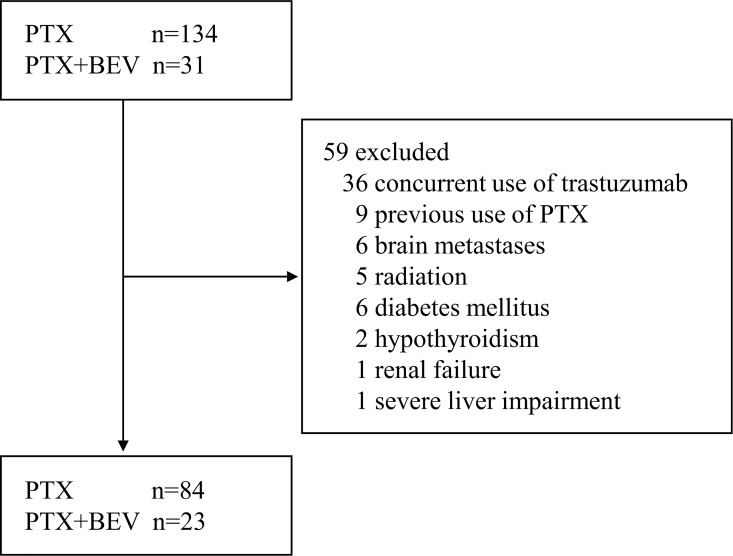
Flow chart of patients through the study. Abbreviations: BEV, bevacizumab; PTX, paclitaxel.

From September 2011 through May 2016, a total of 165 patients with breast cancer received PTX-based chemotherapy, and 107 patients with a median age of 55 (range, 32–83) met the eligibility criteria and were studied (Fig 1). Eighty-four of these patients received PTX, and 23 received PTX+BEV (Table 2). Among the 107 patients, 61 received PTX as adjuvant chemotherapy, 23 received PTX for metastatic disease, and 23 received PTX+BEV for metastatic disease. One patient with human epidermal growth factor receptor-2-positive cancer received PTX alone. There was imbalance in the baseline characteristics between the cohorts: more patients had received previous anthracycline-based chemotherapy in the PTX group than in the PTX+BEV group (*P* = 0.002), and the duration of chemotherapy was longer in the PTX+BEV group than in the PTX group (*P* = 0.001). A total of 34 patients (32%) received treatment for neuropathy: pregabalin in 21 patients (20%), Kampo compounds (Goshajinkgan) in 11 (10%), and vitamin B_12_ in 9 (8%).

**Table 2:** Patient characteristics.

	PTX (n = 84)	PTX+BEV(n = 23)	*P*
Age, years, median (range)	55 (32–83)	57 (42–76)	0.241^†^
Disease state			
Adjuvant	61 (73%)	0	<0.001*
Metastatic/Recurrence	23 (27%)	23 (100%)	
ECOG PS			
PS 0	65 (77%)	14 (61%)	0.342*
PS 1	13 (15%)	6 (26%)	
PS 2	6 (7%)	3 (13%)	
Previous use of docetaxel	13 (15%)	5 (22%)	0.334**
Previous use of anthracyclines	71 (84%)	12 (52%)	0.002*
Positive hormone status	40 (48%)	13 (57%)	0.488*
Previous operation	57 (68%)	17 (74%)	0.620*
Previous radiation therapy	7 (8%)	4 (17%)	0.185**
Treatment for neuropathy	28 (33%)	6 (26%)	0.617*
Duration, courses, median (range)	12 (1–51)	14 (3–42)	**0.001**^†^

†Mann-Whitney test

*chi-square test

**Fisher’s exact test

**Bold letters indicate statistical significance (*P*<0.05).**

Abbreviations: BEV, bevacizumab; ECOG, Eastern Cooperative Oncology Group; n, number; PS, performance status; PTX, paclitaxel.

**Fig 2:**
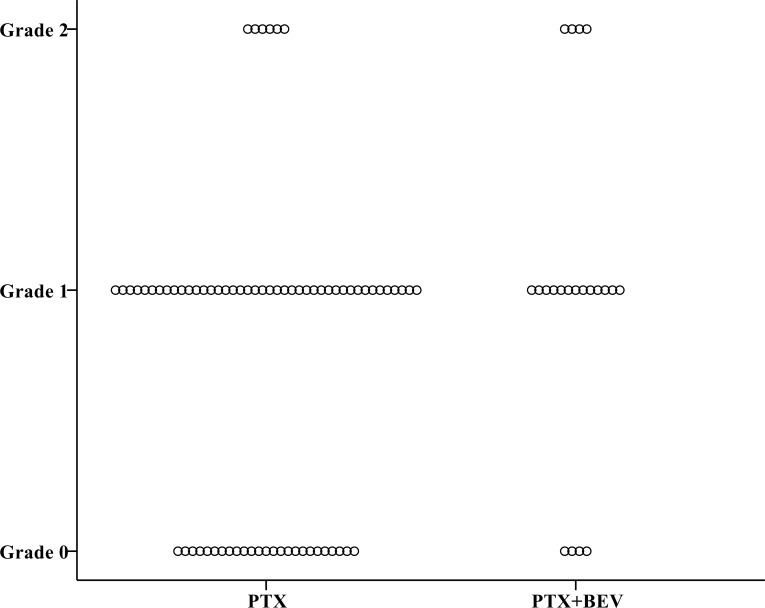
Distribution of CTCAE grades at the 6^th^ course of chemotherapy in the PTX and PTX+BEV groups. CTCAE grade at the 6^th^ course was slightly but not significantly higher in the PTX+BEV group (n = 21) than in the PTX alone group (n = 73) (*P* = 0.095). Abbreviations: BEV, bevacizumab; CTCAE, Common Terminology Criteria for Adverse Events; PTX, paclitaxel.

**Fig 3:**
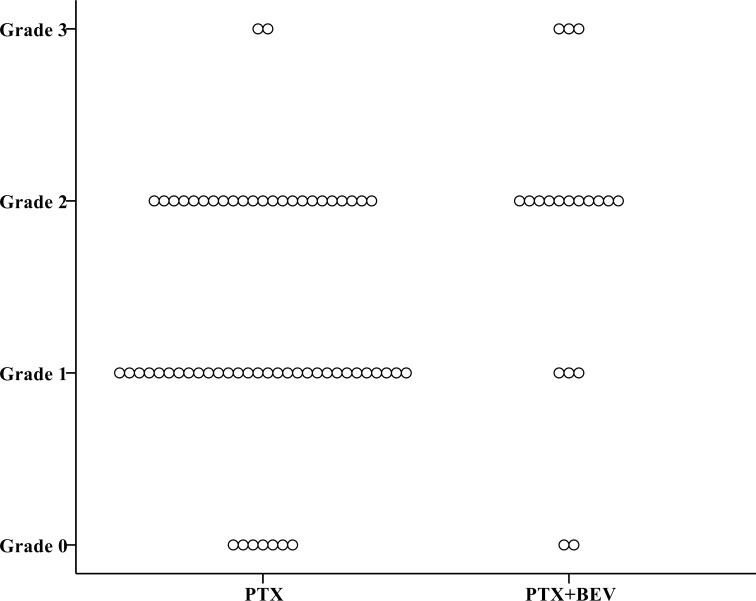
Distribution of CTCAE grades at the 12^th^ course of chemotherapy in the PTX and PTX+BEV groups. CTCAE grade at the 12^th^ course was significantly higher in the PTX+BEV group (n = 19) than in the PTX alone group (n = 62) (*P* = 0.016). Abbreviations; BEV, bevacizumab; CTCAE, Common Terminology Criteria for Adverse Events; PTX, paclitaxel.

**Fig 4:**
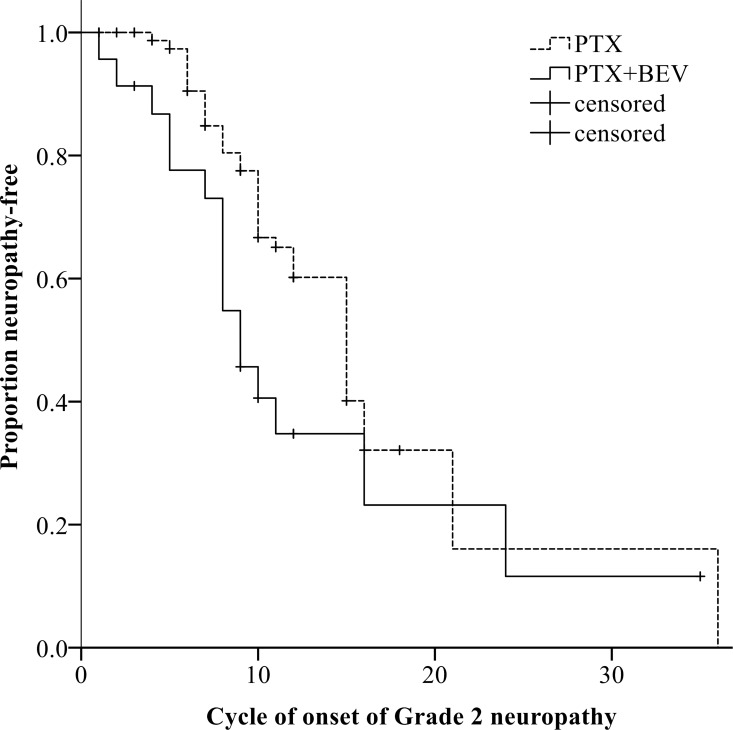
Relation between regimens and time to the onset of Grade 2 neuropathy. In Kaplan-Meier analysis, Grade 2 neuropathy developed significantly earlier in patients given PTX+BEV (solid line) than in those given PTX alone (dashed line) (median number of treatment cycles, 9 vs. 15; *P* = 0.032). Abbreviations: BEV, bevacizumab; PTX, paclitaxel.

Overall, a total of 85 patients (79%) had neuropathy of any grade (Grade 1 in 36 patients, 34%; Grade 2 in 42, 39%; Grade 3 in 7, 7%) (Table 3). At the 6^th^ course, the CTCAE grade was slightly but not significantly higher in patients who received PTX+BEV than in those who received PTX alone (*P* = 0.095) (Fig 2). At the 12^th^ course, the CTCAE grade was significantly higher in the PTX+BEV group than in the PTX alone group (*P* = 0.016) (Fig 3). At the 6^th^ course, the incidence of Grade 2 or higher neuropathy was slightly but not significantly higher in the PTX+BEV group than in the PTX alone group (19% vs. 8%, *P* = 0.154); at the 12th course, the incidence of Grade 2 or higher neuropathy was significantly higher in the PTX+BEV group than in the PTX alone group (74% vs. 40%, *P* = 0.017). Grade 2 neuropathy developed significantly earlier in the PTX+BEV group than in the PTX alone group (median number of treatment cycles, 9 vs. 15, *P* = 0.032) (Fig 4). In multivariate analysis using a Cox proportional-hazards model, which included all variables with *P* <0.20 in univariate analyses, regimen (PTX+BEV) was significantly associated with an increased risk of neuropathy (HR 2.32, 95% CI 1.21–4.44, *P* = 0.012) (Table 4).

**Table 3:** Peripheral sensory neuropathy according to CTCAE.

	PTX	PTX+BEV	*P*
Overall, n (%)	n = 84	n = 23	**0.018**^**†**^
Grade 0	18 (20%)	4 (17%)	
Grade 1	33 (40%)	3 (13%)	
Grade 2	30 (36%)	12 (52%)	
Grade 3	3 (4%)	4 (17%)	
6^th^ course, n(%)	n = 73	n = 21	0.095^**†**^
Grade 0	25 (34%)	4 (19%)	
Grade 1	42 (58%)	13 (62%)	
Grade 2	6 (8%)	4 (19%)	
Grade 3	0	0	
12^th^ course, n(%)	n = 62	n = 19	**0.016**^**†**^
Grade 0	7 (11%)	2 (11%)	
Grade 1	30 (48%)	3 (16%)	
Grade 2	23 (37%)	11 (58%)	
Grade 3	2 (3%)	3 (16%)	
Grade 2 or worse, n(%)			
6^th^ course	6 (8%)	4 (19%)	0.154**
12^th^ course	25 (40%)	14 (74%)	0.017*

†Mann-Whitney test

*chi-square test

**Fisher’s exact test

**Bold letters indicate statistical significance (*P*<0.05).**

Abbreviations: BEV, bevacizumab; CTCAE, Common Terminology Criteria for Adverse Events; n, number; PTX, paclitaxel.

**Table 4:** Multivariate analysis using a Cox proportional-hazard model for the time to the onset of Grade 2 neuropathy.

	Univariate Analysis			Multivariate Analysis		
	HR	95%CI	*P*	HR	95%CI	*P*
Regimen (PTX+BEV)	1.90	1.02–3.51	**0.042**	2.32	1.21–4.44	**0.012**
Age, years	0.99	1.00–1.02	0.46			
Disease state (Metastatic)	1.07	0.57–2.01	0.83			
ECOG PS						
PS 1 (vs. PS 0)	0.34	0.29–1.57	0.37			
PS 2 (vs. PS 0)	0.67	0.69–5.60	0.21			
Previous use of docetaxel	2.06	0.99–4.32	0.055	2.04	0.94–4.41	0.069
Previous use of anthracyclines	1.79	0.85–3.76	0.12	1.62	0.73–3.57	0.24
Positive hormone status	1.06	0.59–1.92	0.84			
Previous operation	1.95	0.87–4.38	0.10	1.87	0.81–4.27	0.14
Previous radiation therapy	1.34	0.58–3.09	0.49			
Treatment for neuropathy^※^	N/A	N/A	N/A			
Duration, courses	0.99	0.95–1.03	0.50			

**Bold letters indicate statistical significance (*P*<0.05).**

^※^Treatment for neuropathy was excluded from this analysis because it was not the cause, but the result of the development of neuropathy.

Abbreviations: BEV, bevacizumab; CI, confidence interval; CTCAE, Common Terminology Criteria for Adverse Events; ECOG, Eastern Cooperative Oncology Group; HR, hazard ratio; N/A, not applicable; PS, performance status.

**Fig 5:**
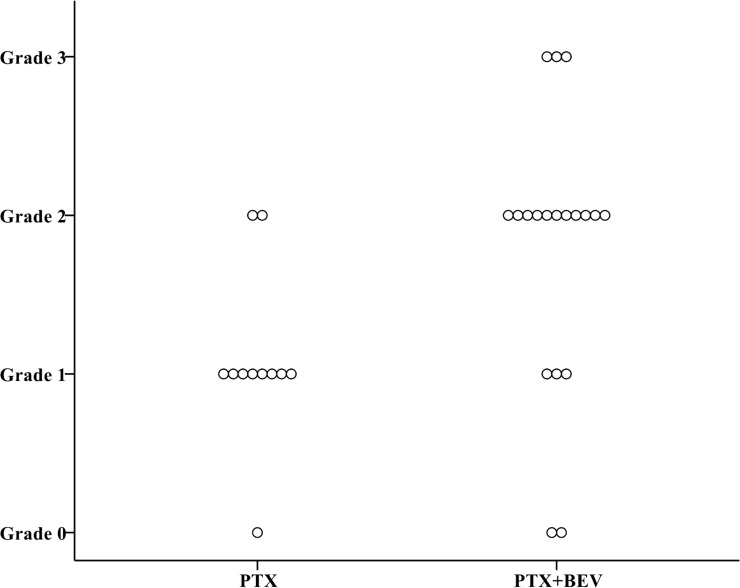
Distribution of CTCAE grades at the 12^th^ course of chemotherapy in the PTX and PTX+BEV groups (metastatic disease). In the subgroup analysis limited to patients with the metastatic disease, CTCAE grade at the 12^th^ course was significantly higher in the PTX+BEV group (n = 19) than in the PTX alone group (n = 11) (*P* = 0.018). Abbreviations: BEV, bevacizumab; CTCAE, Common Terminology Criteria for Adverse Events; PTX, paclitaxel.

**Fig 6:**
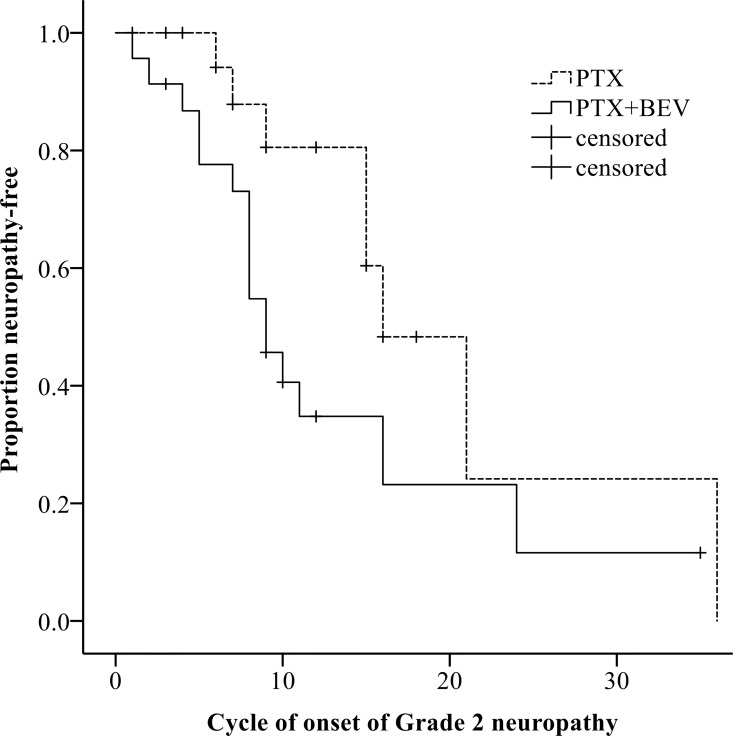
Relation between regimens and time to the onset of Grade 2 neuropathy (metastatic disease). In the subgroup analysis limited to patients with the metastatic disease, Grade 2 neuropathy developed significantly earlier in patients given PTX+BEV (solid line) than in those given PTX alone (dashed line) (median number of treatment cycles, 9 vs. 16, *P* = 0.043) on Kaplan-Meier analysis. Abbreviations: BEV, bevacizumab; PTX, paclitaxel.

The subgroup analysis limited to patients with metastatic disease confirmed similar results (Tables 5 and 6). Particularly in the 12^th^ course, the CTCAE grade and the incidence of Grade 2 or higher neuropathy were significantly higher in the PTX+BEV group than in the PTX alone group (*P* = 0.018, *P* = 0.003, respectively) (Table 6, Fig 5). Again, Grade 2 neuropathy developed significantly earlier in the PTX+BEV group than in the PTX alone group (median number of treatment cycles, 9 vs. 16, *P* = 0.043) (Fig 6).

**Table 5:** Patient characteristics (metastatic disease).

	PTX (n = 23)	PTX+BEV (n = 23)	*P*
Age, years, median (range)	63 (39–83)	57 (42–76)	0.071^†^
Disease state			
Metastatic/Recurrence	23 (100%)	23 (100%)	
ECOG PS			
PS 0	6 (26%)	14 (61%)	0.059*
PS 1	11 (48%)	6 (26%)	
PS 2	6 (26%)	3 (13%)	
Previous use of docetaxel	8 (35%)	5 (22%)	0.326*
Previous use of anthracyclines	13 (57%)	12 (52%)	0.767*
Positive hormone status	17 (74%)	13 (57%)	0.216*
Previous operation	18 (78%)	17 (74%)	0.730*
Previous radiation therapy	7 (30%)	4 (17%)	0.300*
Treatment for neuropathy	5 (22%)	6 (26%)	0.730*
Duration, courses, median (range)	9 (1–40)	14 (3–42)	0.084^†^

†Mann-Whitney test

*chi-square test

**Bold letters indicate statistical significance (*P*<0.05).**

Abbreviations: BEV, bevacizumab; ECOG, Eastern Cooperative Oncology Group; n, number; PS, performance status; PTX, paclitaxel.

**Table 6:** Peripheral sensory neuropathy according to CTCAE (metastatic disease).

	PTX	PTX+BEV	*P*
Overall, n (%)	n = 23	n = 23	**0.029**^**†**^
Grade 0	7 (30%)	4 (17%)	
Grade 1	8 (35%)	3 (13%)	
Grade 2	7 (30%)	12 (52%)	
Grade 3	1 (4%)	4 (17%)	
6^th^ course, n (%)	n = 17	n = 21	0.622^**†**^
Grade 0	3 (18%)	4 (19%)	
Grade 1	13 (57%)	13 (62%)	
Grade 2	1 (6%)	4 (19%)	
Grade 3	0	0	
12^th^ course, n (%)	n = 11	n = 19	**0.018**^**†**^
Grade 0	1 (9%)	2 (11%)	
Grade 1	8 (73%)	3 (16%)	
Grade 2	2 (18%)	11 (58%)	
Grade 3	0	3 (16%)	
Grade 2 or worse, n (%)			
6^th^ course	1 (6%)	4 (19%)	0.243**
12^th^ course	2 (18%)	14 (74%)	0.003*

†Mann-Whitney test

*chi-square test

**Fisher’s exact test

**Bold letters indicate statistical significance (*P*<0.05).**

Abbreviations: BEV, bevacizumab; CTCAE, Common Terminology Criteria for Adverse Events; n, number; PTX, paclitaxel.

## Discussion

To our knowledge, this is the first clinical study to show that the concurrent use of BEV actually exacerbated PTX-induced neuropathy during the same time frame. BEV combined with PTX worsened PTX-induced neuropathy as compared with PTX alone during the fixed period of treatment, independently whether the duration of chemotherapy was extended. Our results suggest that VEGF or VEGF-receptor (VEGFR) inhibitors, including BEV, exacerbate chemotherapy-induced neuropathy as a drug class effect.

Concurrent use of BEV appeared to exacerbate PTX-induced neuropathy in previous clinical studies. For example, in the E2100 trial, in which PTX+BEV prolonged PFS as compared with PTX alone in 722 patients with previously untreated metastatic breast cancer, the incidence of peripheral neuropathy of Grade 3 or higher was significantly higher in patients who received PTX+BEV than in those who received PTX alone (23.6% vs. 17.6%, *P* = 0.03) [2]. In a phase 2 trial performed in Japan, in which PTX+BEV was administered to 120 patients with previously untreated metastatic breast cancer, the incidence of peripheral neuropathy of Grade 3 or higher (31 patients, 26%) was apparently higher than expected [3]. Moreover, in the AURELIA trial, in which BEV combined with chemotherapy (pegylated liposomal doxorubicin, weekly PTX, or topotecan) improved PFS as compared with chemotherapy alone in 361 patients with platinum-resistant recurrent ovarian cancer, the incidence of neuropathy of Grade 2 or higher, analyzed per cycle, appeared to be higher in patients given PTX+BEV than in those given PTX alone from the start to the withdrawal of chemotherapy [8]. The investigators of these studies attributed the increase in neuropathy to the longer period of treatment in patients who received PTX+BEV. However, recent evidence has revealed that endogenous VEGF is strongly associated with the development, protection, and regeneration of sensory neurons [9–11]. In particular, a recent experiment in mice showed that VEGF inhibitors can interfere with neuroprotective effects of endogenous VEGF, potentially triggering the exacerbation of PTX-induced neuropathy [10]. Moreover, delayed wound healing, one of the common class side effects caused by BEV, might hinder the regeneration of peripheral nerves, resulting in severer neuropathy in patients who receive the drug. Our results clearly show that the severity of neuropathy evaluated at the same time points was significantly worse in patients given PTX+BEV than in those given PTX alone.

The exacerbation of chemotherapy-induced neuropathy appears to be a drug class effect of VEGF or VEGFR inhibitors. In the E3200 trial, in which the addition of BEV to FOLFOX4 (oxaliplatin, fluorouracil, and leucovorin) improved survival in 829 patients with previously treated metastatic colorectal cancer, the incidence of peripheral neuropathy of Grade 3 or higher was significantly higher in the BEV arm (16.3% vs. 9.2%, *P* = 0.011) [12]. In the NSABP C-08 trial, which investigated the effectiveness of adding BEV to modified FOLFOX6 (oxaliplatin, fluorouracil, and leucovorin) as adjuvant treatment in 2,710 patients with stage 2 or 3 colon cancer, the incidence of peripheral neuropathy of Grade 2 or higher was significantly higher in the BEV arm (48.9% vs. 43.7%, *P*<0.01) [13]. In these studies, the exacerbation of oxaliplatin-induced neuropathy was attributed by the authors to a longer duration of chemotherapy or higher cumulative dose, leading to increased exposure to oxaliplatin in the BEV arm [12, 13]. However, an *in vitro* experiment showed that a neutralizing antibody against endogenous VEGF exacerbated oxaliplatin-induced neurotoxicity, reflecting the neuroprotective effect of endogenous VEGF [9]. Therefore, similar to the mechanism of PTX, BEV might exacerbate oxaliplatin-induced neuropathy by inhibiting endogenous VEGF [10]. Furthermore, in the RAINBOW trial, in which ramucirumab, a novel humanized monoclonal antibody VEGFR-2 antagonist, in combination with PTX, given as 2^nd^ line chemotherapy, improved overall survival in 665 patients with advanced gastric cancer, ramucirumab was associated with a higher incidence of peripheral neuropathy of all grades than was PTX alone (46% vs. 37%) [14]. Again, the authors attributed the higher incidence of PTX-induced neuropathy to the higher cumulative dose of PTX due to longer PFS in the ramucirumab arm [14]. The higher incidence of PTX-induced neuropathy might have also been ascribed to a class side effect of VEGFR inhibitors (i.e., caused by interference with the neuroprotective effects of endogenous VEGF) [10]. This class side effect is considered clinically important, because augmented neuropathy would impair patients’ quality of life, resulting in early discontinuation of effective chemotherapy. Future trials of VEGF or VEGFR inhibitors combined with neurotoxic chemotherapy should prospectively assess chemotherapy-induced neuropathy between two arms (chemotherapy with or without VEGF or VEGFR inhibitors) at the same time points.

Our retrospective study had several limitations besides a small number of patients. First, the baseline characteristics differed between the cohorts, reflecting the retrospective nature of this study. A prospective study in a larger number of patients would provide a better understanding of this class side effect. Second, we used the number of treatment courses, not the cumulative dose of PTX, to decide the timing for comparing the severity of neuropathy between the cohorts. We chose the number of treatment courses to enable time-to-event analysis, such as Kaplan-Meier and Cox proportional hazards analysis, although use of the cumulative dose of PTX would have most likely provided a more objective assessment of dose-dependent and cumulative effects of PTX on neuropathy. Third, electrophysiological studies and other validated neuropathy severity scales were not used to assess neuropathy in this study.

In conclusion, the concurrent use of BEV was associated with the exacerbation of PTX-induced neuropathy as compared with PTX alone during the same time frame. VEGF or VEGFR inhibitors might exacerbate chemotherapy-induced neuropathy as a drug class effect. Future studies of VEGF or VEGFR inhibitors combined with neurotoxic chemotherapy should prospectively evaluate chemotherapy-induced neuropathy at the same time points, using electrophysiological studies and validated neuropathy severity scales that would provide more accurate information for the evaluation of neuropathy.

## Supporting Information

S1 File Minimal Data Set. (XLSX)
